# High-value breast cancer care within resource limitations

**DOI:** 10.1093/oncolo/oyae080

**Published:** 2024-05-23

**Authors:** Didier Verhoeven, Sabine Siesling, Claudia Allemani, Pankaj Gupta Roy, Luzia Travado, Nirmala Bhoo-Pathy, Clifford Rhayns, Hans Junkermann, Seigo Nakamura, Nwamaka Lasebikan, Forrest Lee Tucker

**Affiliations:** Department of Medical Oncology, University of Antwerp, AZ KLINA, Brasschaat, Belgium; Department of Health Technology and Services Research, Technical Medical Centre, University of Twente, Enschede, The Netherlands; Department of Research and Development, Netherlands Comprehensive Cancer Organization (IKNL), Utrecht, The Netherlands; Cancer Survival Group, Department of Non-Communicable Disease Epidemiology, London School of Hygiene and Tropical Medicine, London, United Kingdom; Nuffield Department of Surgical Sciences, University of Oxford, Oxford, United Kingdom; Champalimaud Clinical and Research Centre, Champalimaud Foundation, Lisbon, Portugal; Department of Epidemiology, University of Malaya, Kuala Lumpur, Malaysia; Just4Cancer, Las Vegas, United States; Mammographie-Screening Neckar-Alb, Tübingen, Germany; Division of Breast Surgical Oncology, Department of Surgery, Showa University, Tokyo, Japan; Department of Radiation and Clinical Oncology, University of Nigeria Teaching Hospital, Enugu, Nigeria; Virginia Biomedical Laboratories, Wirtz, VA, United States

**Keywords:** breast cancer, value quotient, health outcomes, early diagnosis, economics, global health, community outreach

## Abstract

Breast cancer care is a costly global health issue where effective management depends on early detection and treatment. A breast cancer diagnosis can result in financial catastrophe especially in low- and middle-income countries (LMIC). Large inequities in breast cancer care are observed and represent a global challenge to caregivers and patients. Strategies to improve early diagnosis include awareness and clinical breast examination in LMIC, and screening in high-income countries (HIC). The use of clinical guidelines for the management of breast cancer is needed. Adapted guidelines from HIC can address disparities in populations with limited resources. Locally developed strategies still provide effective guidance in improving survival. Integrated practice units (IPU) with timely multidisciplinary breast care conferences and patient navigators are required to achieve high-value, personalized breast cancer management in HIC as well as LMIC. Breast cancer patient care should include a quality of life evaluation using ideally patient-reported outcomes (PROM) and experience measurements (PREM). Evaluation of breast cancer outcomes must include the financial cost of delivered care. The resulting value perspective should guide resource allocation and program priorities. The value of care must be improved by translating the findings of social and economic research into practice and resolving systemic inequity in clinical breast cancer research. Cancer survivorship programs must be put in place everywhere. The treatment of patients with metastatic breast cancer must require more attention in the future, especially in LMIC.

## Teaser

Evaluation of breast cancer outcomes must include the financial cost of delivered care to guide resource allocation and priorities. This article focuses on the value of treatment of patients with breast cancer, globally and especially in low- and middle-income countries.

**Implications for Practice**

A global, coordinated alliance among breast cancer clinicians, advocates, management specialists, and others can optimize the quality of breast cancer care and value across diverse socio-economic, political, and geographic environments. To integrate a value quotient into breast cancer care, we must shift our focus from volumes to values and emphasize patient-centered outcomes as important metrics. Improved clinical outcomes with lower costs may require a transformation of health delivery systems including better organized public health systems with universal health coverage, and the development of public-private partnerships where appropriate.

## Introduction

Undesirable variations in early diagnosis, treatment, and quality management of breast cancer lead to suboptimal outcomes worldwide. Survival varies hugely worldwide.^1^ Breast cancer deaths disproportionately affect women, especially in LMIC. To achieve improved outcomes for all patients with breast cancer worldwide, engagement of all stakeholders and a coordinated approach of their efforts is needed.^2^ Strategies for breast cancer care delivery will be defined. These must be adapted to all socio-economic environments worldwide, reflecting their needs and capabilities, including human and infrastructural resources. Breast cancer can be considered as a global sentinel disease. The principles and structural aspects of an optimal care delivery model, having similar attributes applicable for many other chronic diseases, eg, other malignancies such as colorectal cancer and cervical cancer, diabetes, hypertension, and coronary artery disease, will be discussed. In early 2020, “Breast cancer: Global Quality Care” was published by Oxford University Press.^3^ The reviewers confirmed the absence, but also the need for a more global and comprehensive vision of the disease. The need for a global strategy was supported by the first report of the Lancet Commission on breast cancer,^4^ as well as by the WHO Global Breast Cancer Initiative (GBCI). The purpose of the GBCI is to reduce breast cancer mortality by 2.5% per year. Accordingly, the GBCI focuses on 3 main aspects of breast health: health promotion, timely diagnosis, and comprehensive treatment, including survivorship. Furthermore, we will try to identify the challenges and needs of breast cancer care delivery worldwide.

## Methods

During the last 5 years, a faculty of 150 experts from 5 continents and 30 different countries was established. We communicated during expert meetings, mail, virtual conferences, webinars, and phone conversations to define a collaborative vision on quality breast cancer care. We collected the insights and perspectives of these leading experts including physicians, epidemiologists, economists, pharmacists, psychologists, nurses, patient advocates, journalists, and academics. This project focuses on the melding of high-quality value-based breast cancer care influenced by local economics and resources. The project is continuously under review by the faculty.

The relevant literature was screened from PubMed (MEDLINE), by using the following search terms: “breast cancer,” “value based health care or quality care or quality management or high value care,” and “resource limitations or low-middle income country or low-income country or resource limited country.” The presence of a limited literature was confirmed. Only 17 publications were found, from whom 5 were considered relevant. These findings justify this expert opinion review.

From the international faculty, 11 experts with expertise in value-based health care were selected coming from high-income countries (HIC; D.V., S.S., C.A., L.T., C.R., H.J., S.N., and L.T.) and LMIC (P.R., N.B.P., and N.L.) to discuss more in detail high-value breast cancer care, trying to define recommendations for better care. The different subjects of this review were divided among the experts:

1. Worldwide trends in population-based breast cancer survival were provided by the CONCORD programme (C.A.).
2. A review-based analysis was made about the advantages and outcomes of screening, breast awareness, and clinical breast examination (CBE; S.S., H.J., and N.L).
3. Health care spending data of some LMIC (Nigeria, Malaysia, and India) were provided (N.L., N.B.P., and P.R.) in addition to the World Bank and OECD data (L.T.).
4. Data about breast cancer research in some LMIC were described (N.B.P.).

## Resulting findings

### The burden of breast cancer: a global problem

Surveillance of incidence and survival of breast cancer is key to understand the global picture of this disease, to study disparities, and to improve clinical and patient-reported performance.

Breast cancer is now the most common noncutaneous cancer in 140 of 184 countries in the world.^5^ The 5-year estimated world prevalence was 6.2 million persons in 2012.^6^ North America and Europe account for 9.4% and 25.3%, respectively, of the global number of deaths, although mortality rates are decreasing on both continents. The global proportion of deaths is 41% in Asia, 8.3% in Central and South America, 6% in North Africa and Western Asia, and 9% in sub-Saharan Africa. Breast cancer mortality rates are increasing in these world regions.^7^

Population-based survival for patients diagnosed with breast cancer is a key measure of the overall effectiveness of health systems in detecting and managing the disease. This indicator summarizes the efficiency of early diagnosis, screening, treatment, and the availability of resources and local organization of breast cancer care. Global surveillance of breast cancer survival is meaningful only if we routinely monitor trends to assess whether improvement is being achieved. The CONCORD programme for the global surveillance of cancer survival is an example of such a worldwide initiative.^1^ CONCORD-3 highlighted huge differences in age-standardized 5-year net survival worldwide for women diagnosed during 2010-2014, from 66% in India to 90% or more in the United States and Australia.

Despite being a high-income country, in the United States, age-standardized 5-year net survival was more than 10% lower for Black women than for White women, and this difference persisted over time (76.9% for Black women vs 89.1% for White women, in 2001-2003; 78.4% vs 89.6%, in 2004-2009).^8^

Ethnic and socio-economic inequity is also suggested by the differing Black/White breast cancer mortality rates in the United States, ranging from nearly no difference in Massachusetts and Connecticut (northern states) to more than 1.5 in Louisiana and Mississippi (southern states).^9^

### Early diagnosis: awareness versus CBE versus screening

Survival from breast cancer is stage-dependent and significantly influenced by both early diagnosis and treatment of symptomatic disease, as well as mammography screening programs.

**Figure 1.**
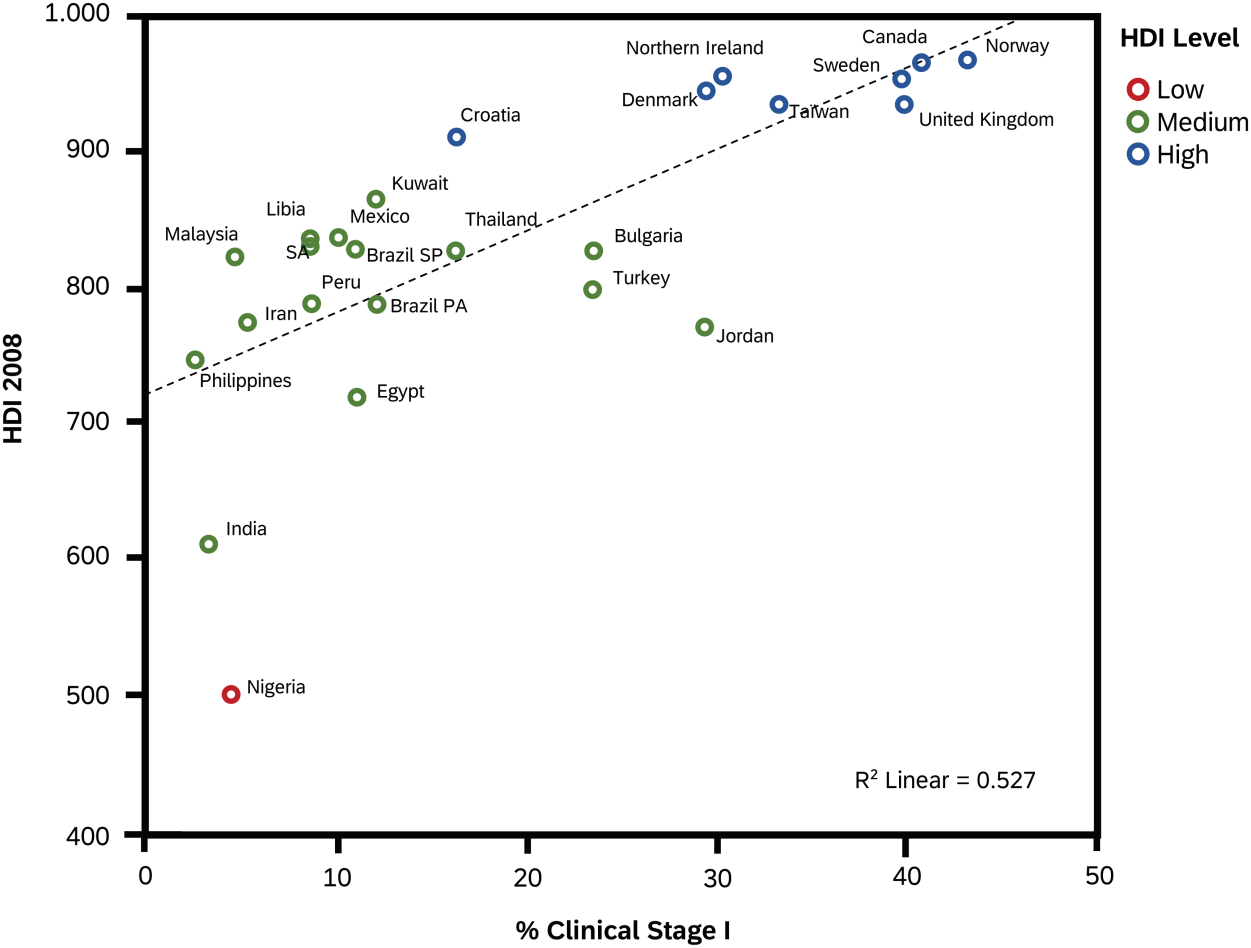
Relation between HDI and Stage I breast cancer Rene Aloisio da Costa Vieira et al.^11^

One of the key quality indicators in the assessment of breast cancer care worldwide is the stage at first diagnosis, which is strongly associated with the human development index (HDI).^10^ The HDI adds the dimensions of life expectancy and education level to national per capita income as a means to stratify a nation’s resource environment (Figure 1,^11^).

**Figure 2.**
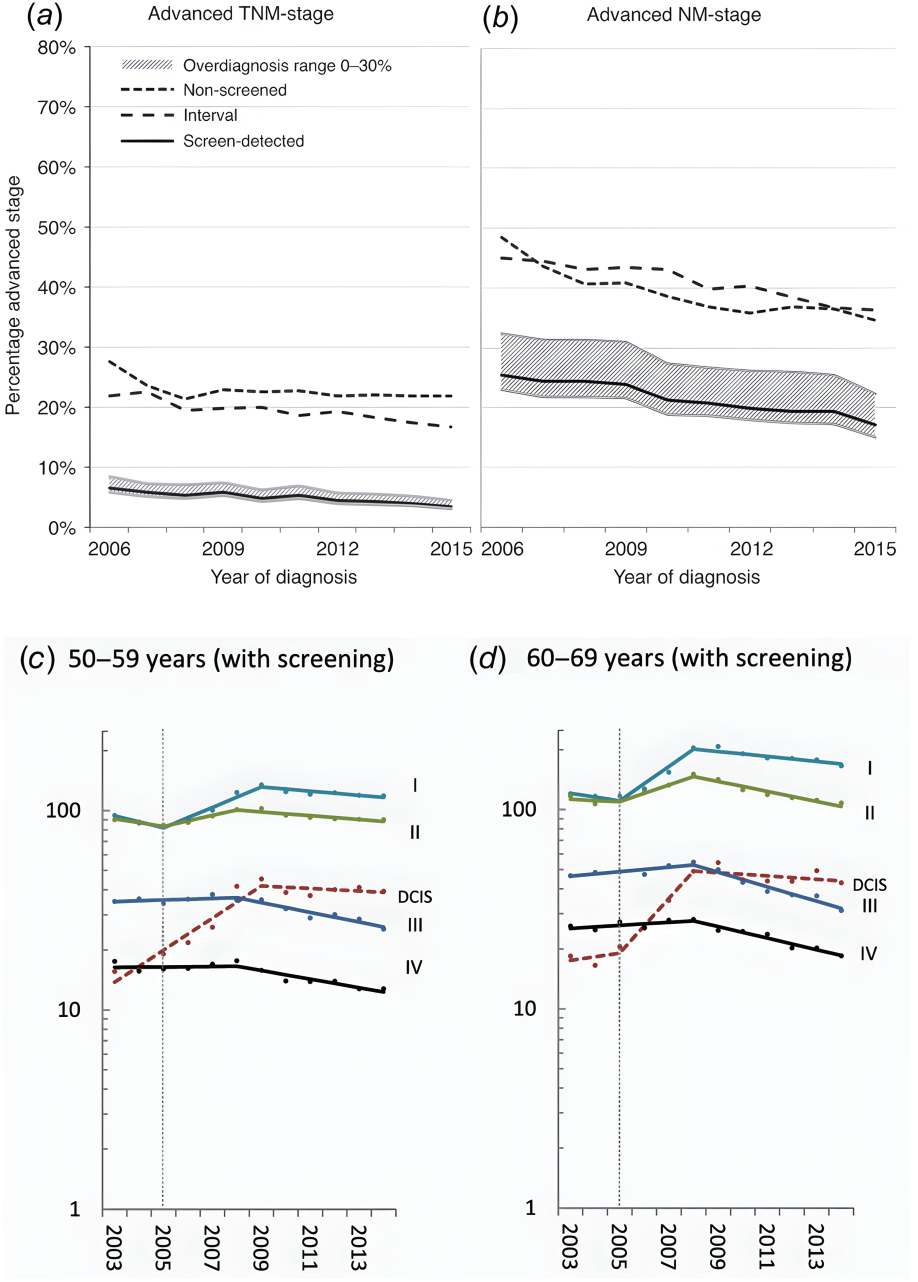
(A, B) Trends in advanced breast cancer incidence in the screen-detected, interval, and non-screened cohorts in The Netherlands. The solid line indicates the screen-detected cancers assuming 10% overdiagnosis. The shaded area indicates the percentage assuming 0% overdiagnosis (lower limit) to 30% overdiagnosis (upper limit; de Munck et al^17^). (C, D) Trends in breast cancer incidence in the screening age groups and stage in Germany. *Y*-axis: age-specific rates/100 000 women on a logarithmic scale. Dots: observed rates, lines rates by join-point regression, vertical dotted line: year implementation of screening (Katalinic et al^19^).

While overviews^12,13^ confirm the effectiveness of mammography screening and international guidelines^14^ reflect the conviction of the large majority of experts that the usefulness of mammography screening has been proven beyond doubt, some questions are occasionally still raised about efficacy and effectiveness.^15,16^ The real challenge is not to find an early disease but to avoid an advanced disease. The most reliable early indicator of the efficacy of a screening program is the reduction of the incidence of advanced-stage cancers in the population offered screening. This is a prerequisite for its effectiveness, leading to a decline in mortality eventually. Recent observational studies have shown the reduction of advanced disease in screening participants in the steady state of a long-term screening program in the Netherlands.^17^ Irrespective of the classification used (TNM or NM),^18^ screen-detected breast cancers were diagnosed less frequently at an advanced stage (Figure 2A, 2B). Also in Germany, after the introduction of mammography screening from 2005 to 2009, a fall of the incidence rate of advanced breast cancers was shown restricted to the target age group (50-69 years; Figure 2C, 2D).^19^ The failure to find such an effect in screening programs of established quality^20^ must be attributed to methodological shortcomings.^21^ In Germany, breast cancer mortality declined in the target age groups, while staying constant in younger or even further rising in older age groups.^19^ The beneficial effect of screening has been maintained under conditions of nowadays Western World breast cancer awareness and advanced modern therapy.

Mammographic screening like any screening for asymptomatic breast cancer also has inherent inevitable negative effects, such as false positive results with cost and stress before a full assessment can exclude malignancy, leading to overdiagnosis, ie, breast cancers which would not have caused complaints, nor have been detected and treated during the remaining lifetime of the individual woman. Moreover, premalignant lesions as DCIS (ductal carcinoma in situ), which have a variable risk of becoming invasive, are detected frequently through the screening programs. The question of overtreatment of low-risk DCIS is currently addressed in several trials (LORIS, LORD, and COMET).^22^ Quality control is of paramount importance to achieve and maintain a positive balance between positive and negative effects of screening programs. Personalized screening is now being tested with the aim of further improving this balance.^23^

Though a recent review has stated that mammography screening would be cost-effective also in LMIC,^24^ especially in the Upper-MIC, mammography screening is mostly not a relevant tool in LMIC due to its complexities, cost, and high infrastructural needs. In addition, a lack of funding might not support widespread national mammographic screening programs.

Clinical breast examination is often regarded as a better option for screening in resource-limited countries. In contrast to the high level evidence on the effectiveness of mammography screening, the evidence for the effectiveness of CBE is still scarce. An overview of meta-analyses of randomized studies on CBE^25^ concluded in 2020: “There is no ‘direct’ evidence (from RCTs which compared CBE with no screening) that CBE is effective in terms of reducing breast cancer mortality.” The Canadian National Breast Screening Study (CNBSS) has been considered by some as indirect evidence of the equivalence of CBE with mammography as a screening method.^26^ Recent evidence, however, documents the long-time suspected corruption of the randomization process in parts of this study invalidating its results.^27^

Results on CBE screening from a randomized trial in Mumbai,^28^ showing that clinical examination of asymptomatic women performed by well-trained personnel may have an effect on breast cancer mortality, should be viewed with caution. Significance was only found in a post hoc analysis of women older than 50 years without any effect in the younger population. The impact would also be restricted because in India, as in most LMIC, the peak of breast cancer mortality is below the age of 50 years.^29^ Recent results from another randomized study of CBE screening in India did not find an effect on breast cancer mortality in any age group although as in the “Mumbai downstaging” was achieved.^30^

In LMIC, the individual status of society regarding education and development of the health care system must be taken into account. Only if the health care system is sufficiently developed to offer adequate treatment for the population and the financial resources are available mammography screening may be an option.^31^

For the majority of LMIC, however, the main priority is to advance diagnosis and treatment of symptomatic disease in order to avoid progression to advanced stages. Delays leading to stage progression have been shown to significantly impair survival.^32^

Since the incidence is rising (see “The burden of breast cancer: a global problem” section), increasing the public awareness of the signs and symptoms of breast cancer is the first step in the implementation of an early detection program. Strategies incorporating breast cancer awareness and equipping health workers with skills to perform quality CBEs potentially play a role in the downstaging of cancers at diagnosis.^33^

Not only financial limitations have to be overcome to reduce the load of advanced breast cancer.^34^ Additional low health literacy, fear and cultural beliefs, high out-of-pocket treatment costs, lack of basic equipment, knowledge, training and skills of health professionals, shortage of specialist staff, difficult access to facilities by over-centralization, and poor communication have been identified in Zimbabwe^35,36^and in the Philippines.^37^ Owing to such factors, 42% of women with suspicious finding on CBE actively refused further assessment in a randomized CBE screening study in the Philippines.^38^ Such factors also have to be considered in certain groups and locations in HIC. Looking more in detail on-screen attendance reveals locales where this indicator is only 5%, even in the United States. Prioritizing resources to identify and screen these population subsets improves the identification of prevalent cancers at an earlier stage (L. Tucker, Virginia, United States, Personal written communication May 16, 2022).^39,40^

### Value-based breast care experience: “more than survival”

Having a diagnosis of breast cancer and undergoing treatment is an emotionally distressing event in a person’s life: it may produce psychological suffering which may impact the patient’s quality of life and survival.^41^ However, distress can be easily screened by the Distress Thermometer,^42^ which is a visual analog scale (from 0 to 10) to rate the level of distress a patient has felt in the past week. It allows for easy screening of psychosocial needs by including a checklist of physical, emotional, social, practical, and spiritual problems. In a landmark study on the prevalence of distress in patients with cancer, 32% of patients with early breast cancer reported high levels of emotional distress,^43^ which increased by 60% in a metastatic phase and continued to increase with the progression of the disease toward the end-of-life.^44^ Severe distress and depression if untreated lead to diminished quality of life, higher clinical complications, shorter survival, and increased health care costs.^45^ Therefore, it is important to screen for the patient’s distress and psychosocial needs early in the treatment pathway to provide adequate psychological support and optimize patients’ well-being and clinical outcomes. This is now considered a quality standard of care for the treatment of patients with breast cancer and a requirement for the European certification of breast units.^46^

Distress management should follow clinical guidelines. A 4-tiered model of professional psychological assessment and support has been recommended to address these needs to organize care in the clinic, which can be adjusted to each country’s available resources. All patients need effective information given through compassionate communication skills to reduce their anxiety related to their disease and treatment, in addition to general psychological/emotional support. This model recommends that this is the first basic level of emotional support which should be delivered by all direct health care professionals (eg, doctors and nurses). Good doctor/provider-patient communication is the essence of higher patient adaptation, higher patient compliance with treatment and care, higher patient satisfaction, and better patient clinical outcomes.^47^ However, for patients with higher levels of distress, the model suggests the involvement of trained professionals in psychosocial oncology care, or mental health professionals, using evidence-based interventions to reduce patients’ emotional suffering.^48^

The recently published essential requirements for quality cancer care of patients with breast cancer ^46^ reinforces the importance of psychologists in the multidisciplinary team in HIC, as well as LMIC, working in an integrated way to assure the patient the best outcomes and quality of life. Patients also require ongoing support in recovering from long and medium-term side-effects of treatments. Cognitive changes, sleep disturbances, fatigue, hot flashes, mood swings, depression, fear of recurrence, self-image alterations, loss of libido and sexual alterations interfere with normal return to work, and resumption of life, family, and social roles. Survivorship care is a much-neglected area and we need to develop programs for better support our patients in resuming a normal life. The European Guide on Quality Improvement in Comprehensive Cancer Control does recommend a survivorship care plan that enhances patient’s self-management and quality of life.^49^

Value Quotient Breast Care may be defined as improved patient-centered outcomes (survival and well-being), following the identification and treatment of a benign or malignant breast abnormality against the costs of full-cycle clinical management. Patient management expenses are directly related to the extent of clinical interventions, which are proportionate to the stage at diagnosis.^50,51^

What is often missed when analyzing the expenses following a cancer diagnosis is that a lack of well-being in patients may lead to higher direct and indirect costs. In many resource-limited settings, the measure of success in cancer care and control is heavily focused on survival. “How well” patients live following a cancer diagnosis, as well as “how long” they live should be acknowledged as important. Patient-centered outcomes such as quality of life and return to work offer important insights into the value of breast cancer care.^52^

Health executives, policymakers, clinicians, and patient advocates must collaborate to design and implement comprehensive breast care services, encompassing the full cycle of breast health from the asymptomatic individual presenting for screening through diagnosis, treatment, supportive care, survivorship phase, and end of life. These initiatives are necessary to address challenges and opportunities to improve breast care value across diverse geopolitical and socio-economic environments.

Common to all scenarios is the desirability of a functioning breast integrated practice unit (IPU) with a focus on performance data collection and informed decision-making for all aspects of service and patient outcomes. The establishment of breast IPUs with a regular, (at least weekly) multidisciplinary breast cancer planning conference must be considered a major public health achievement.^53^ This team evaluates patient-specific disease attributes to recommend the best, personalized, and cost-effective diagnostic and treatment options. Irrespective of whether the clinical environment is HIC or LMIC, the IPU must adopt a culture of performance measurement with the adoption of clinically and financially relevant and actionable metrics. Clinical and financial outcome measures must be regularly and critically reviewed by clinicians and managers empowered to improve performance.

Patient navigators serve an essential role in the improvement of the patient experience and outcomes as patient advocates in the multidisciplinary team.^54^ Navigators are well-positioned to coordinate the care of individual patients. When provided with clinical practice protocols, navigators can facilitate the diagnostic workup and reduce or eliminate over- and under diagnosis. Similarly, by coordinating care across the care continuum, including medical genetics, surgery, reconstruction, medical and radiation oncology, and survivorship, navigators can assist in the reduction of both over-and under treatment.

**Figure 3.**
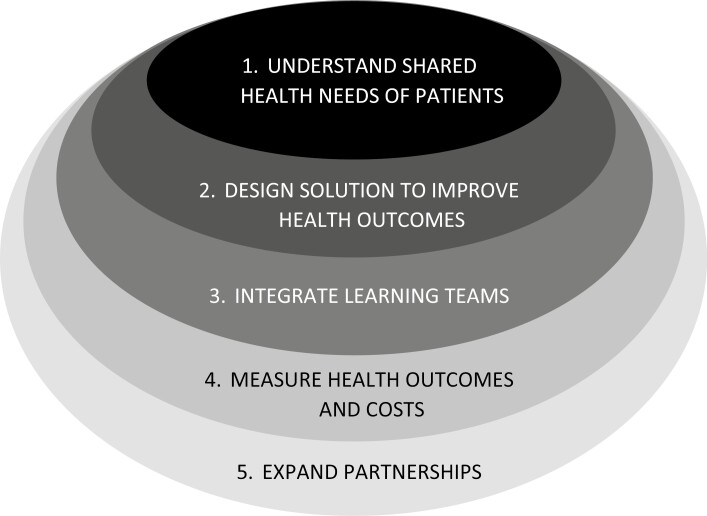
Value-Based Health Care framework, from Teisberg^56^.

Based on the Value-Based Health Care strategic framework defined by Porter^55^ and Teisberg,^56^ the following key steps are proposed for implementation (Figure 3):

1. The clinical and psychosocial needs of patients with breast cancer must be understood.
2. Coordination of care in IPUs to improve performance from community screening to survivorship and end-of-life care:
  ◦ in breast care, accomplished with comprehensive, interdisciplinary centers;
  ◦ multidisciplinary discussions must be mandatory with expertise of different disciplines.
3. Integrated learning teams must be formed:
  ◦ in breast care, this includes marketing, information technology, administration, social services, and rehabilitation;
4. Managing costs: how do we reduce the cost of provided care?
  ◦ in many LMIC, costs are often perceived as too high, influencing the decision to seek care. Many patients are paying for care out of pocket, with the added costs becoming unbearable;
  ◦ begin with stage shifting through clinical and mammographic screening (reduced reliance on tertiary care);
  ◦ imaging-guided biopsy replacing surgical (especially in LMIC).
5. Expanding better organized public health systems/extending the influence of clinical breast centers:
  ◦ providing guidance to breast cancer advocacy groups, payers, and policymakers;
  ◦ social media;
  ◦ discussing the opportunities and pitfalls of public-private partnerships;
  ◦ integration of radiology, pathology, surgery, and oncology clinical services;
  ◦ extend local hospital or clinic services to expand community health initiatives (merging of local breast units or centers into regional health systems with the ability to serve large geographic regions and leverage resources).

### Limitations of global breast care delivery

**Figure 4.**
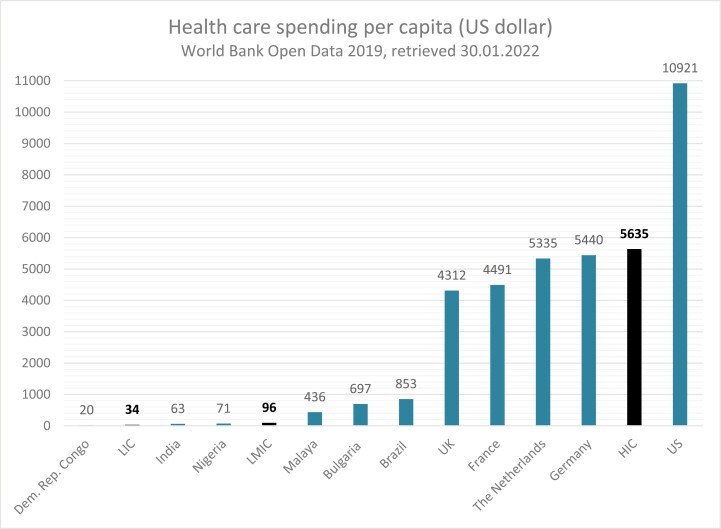
Health care spending per capita US dollar (World Bank Open data, 2019, retrieved January 30, 2022).

Health care spending per capita by source of funding varies considerably among HIC,^57^ LMIC, and LIC as demonstrated in Figure 4. The understanding of the cost in LMIC is limited but critical to guide effective delivery strategies.^58^ There has been nevertheless substantial growth in the number of breast cancer economic evaluations in LMICs in the past decade. The per capita health care spending in 2019 was reported by the World bank database (www.dataworldbank.org) to be 34 in LIC, 96 in LMIC, and 5635 in HIC.^59^ In Malaysia, health financing is largely subsidized by public funding approximating 51% of total health spending. An important issue is out-of-pocket expense and medical care, which is not reimbursed by insurance or government payments. In 2020, this out-of-pocket spending soared to 43% of total health spending in the nation. Nigeria’s health spending per capita remains even lower but with an out-of-pocket expenditure on health estimated at three-quarters of the nation’s health expenditure in 2018. A large percentage of the population is unable to afford—and have limited access to—cancer treatment services.^60^ In LMIC, and even in some HIC, a breast cancer diagnosis can be a financial catastrophe with important bill problems for the patient and family. Medical bill problems are defined as unexpected insurance denials, co-pays, deductibles, or out-of-pocket expenses.^61^ The pooled rate of financial toxicity for patients with breast cancer was 78% in low- and middle-income countries and 35% in HIC.^62^

Societal health care expenditures, of which a considerable part is due to breast cancer care, are rising to levels that may not be sustainable in the future. Cancer causes high costs both within and outside the health care system, in part due to the rising cost of cancer drugs. Economic evaluations of new and existing therapies can be used to inform budget allocations in a way that maximizes health outcomes and broader value to the patient. It is increasingly recognized that personalized care, defined as a better selection of those patients getting most advantage of treatment, can offer more value for patients and at the same time provide value for money. It is timely that current clinical practice guidelines are revisited toward this personalized approach, acknowledging the patient’s voice, as well as the cost to society of therapy. The best example is the correct identification of the receptor and Her2 status. These data are many times missing in LMIC. Another example is the recognition of the health assessments to identify the appropriateness of therapy and the calculation of the survival gain of different treatments. The possibility of de-escalation of 12- to 6-month adjuvant trastuzumab can be considered as an example of decision-making in a resource-limited setting, but hampered by the difficulty in changing international guidelines. A 12-month regimen of trastuzumab had an additional annual cost of US$6 million in Peru, being too expensive considering the limited budget.^63^ Hypofractionation of radiation therapy, from 25 to 5 fractions is another example of introducing cost-efficiency.^64^ The use of clinical benefit scales of the American Society of Clinical Oncology (ASCO) and the European Society of Medical Oncology (ESMO) are considered valuable tools but have not gained enough acceptance.^65,66^

Breast cancer care is hampered by numerous local circumstances, including variations in availability and access to resources, administrative efficiency, and organization of the care process. The differences in access are related to local affordability and timeliness of care. Timeliness is defined by wait time for appointments and time to obtain information and reports. Affordability means the ability to pay for the care, such as having an insurance to cover the expenses with minimal out-of-pocket cost. Significant disparities are reported by the OECD in the health care performance even among different HIC (https://worldpopulationreview.com/country-rankings/cancer-survival-by-country). National strategies and policies should ideally be inspired by the best available models to reduce the financial burden of a breast cancer diagnosis. Tailoring clinical practice guidelines to the local context results in a valuable, resource-efficient tool that can be used by health professionals and patients to focus on ethnic differences and the assumption of different biologic behavior between different races and nations.^67,68^ An example is the collaboration between ESMO and the Japanese Society of Medical Oncology started in 2016, becoming an active partnership between the oncology societies of China, India, Indonesia, South Korea, Malaysia, Philippines, Singapore, Taiwan, and Thailand integrating ethnic, scientific, socioeconomic, and local practice characteristics.^69^

Evaluation of breast cancer care must also give special attention to the reduction of “avoidable deaths,” defined in this context as deaths from all causes that are considered to have been due to medical or laboratory errors. Important differences remain among OECD members states not only in the proportion of avoidable deaths (mean of 199 deaths per 100 000 population in OECD countries, ranging from 139 in Australia, 191 in Chili, 216 in Turkey, 265 in United States to 366 in Mexico) but also in each country’s success in reducing these deaths.^70^ A poor performance suggests a worse access to primary care, prevention, and chronic disease management.

### Clinical breast cancer research in vulnerable populations

A look at the world map of clinical breast cancer studies showed that in 2022, only 246 clinical trials were registered in Africa, 388 studies in South America, 161 in India, and 269 studies in Southeast Asia, compared to 3135 studies in Europe.^71^ In contrast, there appears to be a rapid increase in China with 1635 clinical trials registered in 2022. Enrollment in clinical trials must reflect the demographic diversity of people of the health condition under study. Barriers to the participation of marginalized communities must be removed.^72^ In addition, more attention must go to include members of ethnic minorities, people with disabilities and geriatric populations. Initiatives from all parties in clinical and translational research are needed to translate biomedical discoveries into health equity for all. The right questions must be asked of the representative patient populations to receive the right answers. More attention must go to real-world evidence with attention to benefit and risk derived from the analysis of real-world data. In their recent draft guidance, the US Food and Drug Administration (FDA) discusses the use of real-world data in support of decision-making about the safety and efficiency of new drugs.^73^ Electronic health records, medical claims data, and patient registries must all be evaluated. Breast cancer management strategies in the LMICs must not adopt but rather adapt Western knowledge as most of the current knowledge on breast cancer has been generated in Western populations. As an example, socio-economic profiles, life style, culture, and genetic background of Asian and Western women are substantially different from each other.^74^

An illustrative example is the research on artificial intelligence with thermal images of early breast cancer developed in India.^75^ The proportion of dense breast is almost twice in Asians and Africans compared with Caucasians. Therefore, classical mammography screening might be less adequate for Asians and Africans.

Indirect cost rate contributes importantly to research inequity in global health research. Additional funding for this cost can provide critical support for infrastructure and facility operations fueling the capacity to conduct more research in LMIC. Discussions with international research partners on how to use investments more adequately could strengthen global research.^76^ In addition, more studies must focus on survivorship and patient-centered outcomes in these populations.

## Discussion

### Breast cancer: a global problem

Breast cancer is a major global health problem with increasing incidence, especially in LMIC. Monitoring worldwide survival trends is a key to formulating strategies for global breast cancer control, as shown by the CONCORD programme.

#### Early diagnosis: awareness versus CBE versus screening

In HIC, mammographic screening of the asymptomatic population has been effective in shifting diagnosis to an earlier stage. The critical issue of obtaining less advanced-stage breast cancer after the introduction of a screening program must be established. Strategies to improve cancer detection in LMIC should emphasize the development of national breast cancer networks to coordinate care and to promote clinical early detection. Efforts to increase early detection strategies should accompany those to increase access to treatment. As such, costly community mammographic screening detection initiatives may be a priority only when implemented following the deployment of clinical evaluation protocols for symptomatic and asymptomatic individuals with available access to quality treatment. Treatment facilities must be strengthened to accommodate the accompanying volume as most treatment facilities in LMIC have limited human and infrastructural capacity, with fragile health systems that can be easily overwhelmed. In LMIC, cultural, economic, and logistic barriers may render mammographic screening an inefficient method for the early detection of breast cancer. Findings about CBE from a large, randomized clinical trial in Mumbai are viewed with optimism by the global cancer community. The situation in each country must be analyzed individually before an action plan can be implemented. All breast cancer care activities must be developed in a coordinated pattern to achieve the desired results avoiding low-value or harmful practices.^77^ The WHO’s GBCI develops resource-stratified guidelines for the implementation of early detection and therapy of breast cancer programs.

#### Value-based breast care experience: “more than survival”

Strategies to improve high-value breast cancer care have to be defined in HIC, but even more in LMIC. These strategies for care delivery must be scalable and appropriate for diverse socio-economic environments worldwide, reflecting the different needs of LMIC and HIC. The principles and structural aspects of an optimal care delivery model include specialized clinical leadership, regularly updated clinical guidelines, multidisciplinary coordination of care, and rigorous measurement of clinical and value quotient outcomes including PROMs and PREMs. The value should be defined around the patient with breast cancer, in a well-functioning health care system. The creation of value could even determine the rewards available to care providers. The multidisciplinary breast cancer conference is considered to be “the jewel in the crown” of the IPU, coordinating multiple specialties and functions around patients with breast cancer. Their task is to define personalized treatment opportunities for shared decision-making with the patient discussing the best opportunities taking into account available resources.

#### Limitations of global breast care delivery

Financial toxicities among patients with breast cancer are substantially higher than among other health conditions. While data on spending for breast cancer care are largely unavailable in the LMICs, it is conceivable that lack of funding for health care and rising OOP spending in these countries will have a detrimental impact on cancer care delivery and the financial well-being of households affected by breast cancer. Innovations such as precision medicine may help reduce over- and under treatment but must be evaluated from a rigorous value perspective. The costs associated with specific diagnostic studies and treatments hamper access: drugs do not benefit those who cannot afford them. Transparent, fair, and evidence-based decision-making with a value quotient perspective must guide the allocation of our limited resources to achieve high-quality care. Entry agreements with pharmaceutical companies may be used to manage risks when a therapy lacks supportive clinical evidence. The value perspective must also be used to support shared decision-making by integrating patient-reported outcomes, clinical evidence, and broader societal considerations. Equity in breast cancer care must be ensured for all patients with breast cancer. Justice can only be provided by fixing the system to offer equal access in LMIC but also in HIC to both tools and opportunities.^78^ There is an urgent need for more resources to aid early detection and provide financial protection from the cost of a cancer diagnosis. Utilizing existing community platforms such as HIV awareness programs could also improve breast cancer awareness and CBE practices.

#### Periodical comparison of key quality indicators

Identification and reporting of some key quality indicators are a minimal requirement in HIC and LMIC. Most of them were suggested by the Breast Health Global Initiative (quality indicators 2, 4 and 5):

1. real-world population-based survival estimates, as shown by the CONCORD programme;
2. stage distribution at first diagnosis, with a minimum level of 60% of stage I or II (BHGI data);
3. distribution of ER, PgR, and HER2 status examination;
4. diagnostic interval of maximally 60 days between first observation and the start of therapy (BHGI data); and
5. 80% of patients who accomplished the proposed treatment (BHGI data).

#### Clinical breast cancer research in vulnerable populations

There is a clear need for research in vulnerable populations adapted to the local environments, taken into account ethnic differences, local resources, and local organization of breast cancer care. Apart from intervention studies, diagnostic and prognostic studies are also local-specific and have to be validated in LMICs before implementation in their routine clinical practice.

## Data Availability

No new data were generated or analyzed in support of this research.
